# Health-related quality of life and impact of socioeconomic status among primary and secondary school students after the third COVID-19 wave in Berlin, Germany

**DOI:** 10.1371/journal.pone.0302995

**Published:** 2024-05-09

**Authors:** Mascha Kern, Toivo Glatz, Marcus A. Mall, Joachim Seybold, Tobias Kurth, Frank P. Mockenhaupt, Stefanie Theuring

**Affiliations:** 1 Institute of Public Health, Charité–Universitätsmedizin Berlin, Corporate Member of Freie Universität Berlin and Humboldt-Universität zu Berlin, Berlin, Germany; 2 Institute of International Health, Charité Center for Global Health, Charité–Universitätsmedizin Berlin, Corporate Member of Freie Universität Berlin and Humboldt-Universität zu Berlin, Berlin, Germany; 3 Department of Pediatric Respiratory Medicine, Immunology and Critical Care Medicine, Charité–Universitätsmedizin Berlin, Corporate Member of Freie Universität Berlin and Humboldt-Universität zu Berlin, Berlin, Germany; 4 Berlin Institute of Health (BIH) at Charité–Universitätsmedizin Berlin, Corporate Member of Freie Universität Berlin and Humboldt-Universität zu Berlin, Berlin, Germany; 5 German Centre for Lung Research (DZL), Associated Partner, Berlin, Germany; 6 Medical Directorate, Charité–Universitätsmedizin Berlin, Corporate Member of Freie Universität Berlin and Humboldt- Universität zu Berlin, Berlin, Germany; University of Tartu, ESTONIA

## Abstract

In the earlier phases of the COVID-19 pandemic, studies in Germany and elsewhere found an overall reduction in health-related quality of life (HRQoL) among students. However, there is little evidence on later pandemic stages as well as socioeconomic influencing factors. We aimed to (1) describe HRQoL in a Berlin student cohort at two time points in mid-2021, and to (2) analyze the effects of household income and education. We assessed HRQoL of students from 24 randomly selected primary and secondary schools in Berlin, Germany, with the KIDSCREEN-10 index in June and September 2021. To adjust for non-response bias, inverse probability weighting was applied. The potential effects of both household income and education (lower vs. higher) were estimated in generalized linear mixed models, based on prior assumptions presented in directed acyclic graphs. Our cohort comprised 660 students aged 7–19 years. In June 2021, 11.3% [95% CI = 9.0% - 14.0%] reported low HRQoL, whereas in September 2021, this increased to 13.7% [95% CI = 11.1% - 16.5%], with adolescent girls more frequently reporting low HRQoL at both time points (20% [95% CI = 17.1% - 23.3%] and 29% [95% CI = 25.5% - 32.5%]) compared to boys and younger children. While there was no statistically significant total effect of lower household income on HRQoL, a negative effect of lower household education was statistically significant (*β* = -2.15, SE 0.95, 95% CI = -4.01 to -0.29, *p* = 0.024). In summary, students’ HRQoL in mid-2021 was better than that documented in other studies conducted at pandemic onset using KIDSCREEN-10. Female adolescents reported low HRQoL more often, and lower household education significantly reduced children’s HRQoL. Support strategies for psychosocial wellbeing should consider socioeconomically disadvantaged children as important target groups.

## Introduction

The Coronavirus Disease 2019 (COVID-19) pandemic has impacted the health and wellbeing of children and adolescents worldwide. While most children present with mild symptoms of COVID-19 or are asymptomatic, the indirect impacts of the pandemic and the public health responses on young people have been considerable [1]. After the World Health Organization (WHO) classified the COVID-19 outbreak as a pandemic in March 2020, many countries took extensive containment measures. Social distancing rules, lockdowns, and school closures were implemented, leading to rapid and widespread changes in the daily lives of students that are reflected in their mental health and health-related quality of life (HRQoL) [2–7]. Dimensions of HRQoL relevant to children and adolescents include self-perception, relationships with parents and friends, as well as school-related wellbeing, and identifying determinants of HRQoL can provide important knowledge for the early detection of support and health care needs [8].

Several studies suggest that household socioeconomic status (SES), either as a composite score or as single components thereof, affected children’s and adolescents’ HRQoL during the COVID-19 pandemic [2–5, 9–12]. While research prior to the pandemic has identified SES or components of it as an essential determinant of HRQoL [13–17], there is a lack of research examining factors influencing low HRQoL during the COVID-19 pandemic [2, 3, 14, 15]. Evidence predominantly from the early pandemic stages showed that pre-existing social inequalities exacerbate inequitable health outcomes as the pandemic progresses, due to unequal experiences of job and financial instability, overcrowding and limited access to green space in lower SES neighborhoods and disruptions of social contact and physical activity [2, 3, 18, 19]. Examining socioeconomic variables as potential causes for differing health outcomes during the pandemic is therefore critical to better understand the underlying mechanisms, identify groups at risk, and improve targeted prevention measures. There are multiple possible causal pathways through which socioeconomic background may affect the outcome HRQoL, including disparities in access to material and social resources, healthcare services, and differential exposure to stress and discrimination [5, 20], and exact mechanisms between single socioeconomic indicators and physical and mental wellbeing are not well understood.

Cross-sectional and longitudinal studies in Germany found an overall reduction in HRQoL in children and adolescents during the initial phase of the COVID-19 pandemic in 2020 [3, 7, 11, 21]. The longitudinal population-based COPSY study (COVID-19 and PSYchological Health Study) assessed HRQoL and mental health of 2,097 children and adolescents in Germany at three consecutive time points between May 2020 and October 2021. Low HRQoL was reported by 40.2%, 47.7%, and 35.1% of participants, respectively, compared to 15.3% prior to the pandemic [4, 22]. In the longitudinal Berlin Corona School Study (BECOSS), where four cross-sectional surveys investigated COVID-19 infection dynamics, as well as sociodemographic, behavioral, and psychosocial parameters among students in Berlin, Germany [23–25], HRQoL was assessed at the end of March 2021, reporting low HRQoL in 44.3% of the children [24]. Yet, studies describing the trajectories of psychosocial wellbeing of students in Germany at later stages of the COVID-19 pandemic are still scarce [26, 27] and lacking for the city of Berlin.

As a part of the longitudinal cohort study BECOSS 2 [28], the present study aimed at (1) describing the distribution of HRQoL in students on two occasions in June and September 2021 and (2) identifying socioeconomic determinants of HRQoL within the cohort. Given that the socioeconomic exposures are constant within the short observation period, we pooled data from both time points for aim 2. We hypothesized that lower household income and lower household education had a negative effect on HRQoL of schoolchildren.

## Materials and methods

### Study design and setting

The observational study BECOSS 2 collected data at 24 Berlin primary and secondary schools at three time points in 2021 [28]: June 21–23 (T1), August 9–11 (T2), and September 27–29 (T3). The present analysis focused on T1 and T3. At T1, Berlin schools had just returned to their original class sizes in early June after a model of split classes (half the original size alternating weekly between distance learning and school attendance) had been in effect since mid-March. School attendance was not yet mandatory. T1 took place directly before the summer school vacation (June 24—August 6, 2021). At T3, students had attended school for six weeks in full classroom sizes under almost normal schooling conditions, wearing masks in classrooms and testing twice weekly for SARS-CoV-2 [29].

For random school selection, the twelve Berlin city districts were first divided into three socioeconomic strata, “low”, “middle”, and “high” [30]. Within each stratum, two districts were randomly selected. All public schools from the selected districts were then put in random order and approached in that sequence until two primary and two secondary schools from each SES stratum agreed to participate [23]. Three of the targeted schools declined to participate and were replaced by substitutes of the same stratum. The resulting 24 schools chose one or two classes depending on factors like teachers’ willingness and disposability regarding exams or school trips during the study period. We aimed to examine 20 students per class. However, the actual number of participating students per class varied from 1 to 28.

As described elsewhere [28], study information and consent forms were sent to the participating schools and distributed to the selected classes before the start of data collection. After the children and their guardians consented to study participation, paper-based questionnaires with instructions for completion were mailed to the home address of the enrolled participants at each study time point. Parents were asked to answer questions on the socioeconomic status of the household in a designated section on the first page of the questionnaire. For the participants in primary schools, parents were also encouraged to help their children with the questionnaire.

### Measures

The questionnaire had a total of 54 items. Questions about the socioeconomic background were directed to parents, children were asked about their sociodemographic data, school environment, leisure time activities, and COVID-19 related circumstances like symptoms, infections, vaccinations, fear and risk of infection as well as health behavior such as wearing of masks, washing hands, and social distancing. As the numbers of infections in the sample were very low, the present study does not include an analysis of these variables. Besides HRQoL, the BECOSS 2 study assessed general anxiety disorder symptoms using the GAD-7 instrument which was the subject of another study [28]. The variables relevant to the present study were assessed as follows.

Household income (four categories: up to 1000€, 1000–2000€, 2000–5000€, more than 5000€) was defined as the average monthly net household income reported by the parents. Due to the small number of observations, we combined the three lowest categories into a “lower income” category. The “higher income” category of more than 5000€ corresponds to at least double the average monthly net income of a Berlin household with one working adult in 2021 [25]. Household education (four categories: elementary school or comparable; secondary school or comparable; higher education entrance qualification; university degree) was assessed as the highest level of completed education in the household reported by parents. The lowest two and the highest two levels were merged, respectively, resulting in a dichotomous categorization (“lower” versus “higher education”).

Additionally, we measured exact age (in days) calculated from birth dates and defined two age categories for comparison with other studies, following the reference norm data from the KIDSCREEN-10 instrument with a cutoff age of 12 years (younger = 8-11 years vs. older = 12-18 years). We documented sex, offering the three options female, male, or diverse. Family migration background (yes, no), answered by the parents, was defined as at least one parent born outside Germany, and household size (four categories: one, two, three or four, five and more persons) as the number of persons living in the same household in addition to the participant.

We assessed HRQoL using the KIDSCREEN-10 self-report Index questionnaire. Inquired fields include physical activity and fitness, moods and emotions, participation in social activities, relationships with parents and peers, as well as the perception of own school performance, referring to the last week prior to filling in the questionnaire [31]. The tool consists of 10 items (e.g., “Thinking about the last week, did you feel fit and well?”), each answered on a 5-point Likert scale from 1 (not at all) to 5 (extremely) or 1 (never) to 5 (always). The scores from 1 to 5 were summed up to raw scores ranging from 10 to 50 and transformed into Rasch person parameter estimates. Subsequently, T-values were computed based on a pre-pandemic European sample with a mean score of 50 and a standard deviation (SD) of 10. Higher values indicate a higher HRQoL. The classification of T-values into categories of low, medium, and high HRQoL was carried out in reference to pre-pandemic European norm data for children and adolescents aged 8–18 years (without accounting for age and sex). Scores half a standard deviation below the population mean were categorized as “low HRQoL”, and half a standard deviation above as “high HRQoL” [32]. Scores were also calculated for responses with one missing value based on alternative norm data. For participants younger than eight and older than 18, no HRQoL category could be computed, and they were therefore excluded from the descriptive analyses. Furthermore, given that the instrument includes two questions directly aimed at last week’s school experiences, these items were left unanswered by many participants at time point T2 directly after school vacation. We decided to exclude T2 data from analyses, because the KIDSCREEN-10 Index could not be evaluated for this time point.

### Data analysis

The paper-based questionnaires were digitized by FormPro Software (OCR Systems, Version 3.1) and transformed into an Excel database. Data were analyzed using R (version 4.2.0) and RStudio (version 2022.7.2.576).

To account for non-response bias and non-random attrition, inverse probability weighting (IPW) was used [33]. Participants were inversely weighted separately at each of the two time points based on their age, sex, and for T3 by participation at T1. These weights were then multiplied across both time points so that children with complete observations were up-weighted to count for children of the same age and sex with missing data. Children who only responded at T1 or T3 were downweighted to 0. The result is an IPW-population, referred to as “pseudo-population”, simulating a situation in which all individuals responded at both time points. All analyses were conducted within the pseudo-population, based on the weighted responses of children and adolescents with complete observations in T1 and T3 to account for missing data from non-respondents of the same age and sex. Additionally, we conducted a sensitivity analysis for the IPW-population including age, sex, school city district, and participation at both time points and provide the results in the (S1 and S2 Files and S1 Table).

To depict the main characteristics of the study population, means and standard deviations were calculated for continuous variables, and categorical variables were presented as proportions. In addition, means and standard deviations of HRQoL (T-values), as well as frequencies and percentages of HRQoL categories (low, medium, high) were presented stratified by time point, sex, and age group.

For the analysis of the causal research questions, we followed the approach as proposed by van Zwieten et al. [34]. Assumptions on the underlying relations between the two exposures (income, education) and the outcome (HRQoL) with all potentially relevant variables from the BECOSS 2 dataset were depicted graphically using directed acyclic graphs (DAGs) separately for each effect [35, 36] (S1 and S2 Figs). Variable selection and construction of the causal diagrams to establish potential confounding and mediating variables were based on subject matter knowledge and previous research on causal relationships of SES (or income and education separately) and health outcomes [5, 9, 17, 20, 37–40]. Minimal adjustment sets for variables potentially leading to confounding according to the causal diagram were determined using the software DAGitty [41]. Given the aim of estimating the average total causal effects, mediators were not adjusted for and therefore omitted from the DAG [34].

Two generalized linear mixed models were set up to estimate the average total causal effects of household income and education on HRQoL separately. To increase the precision of the estimate, both waves of observations were included per participant. The model, therefore, provides an average estimate over the two time points which makes it more independent of pandemic-related events like lockdowns, split classes, and school holidays. The first model used T-values of HRQoL as the dependent variable and household income as well as the potential confounding variables household size, education, and family migration background as independent variables. The second model used T-values of HRQoL as the dependent variable and household education plus the potential confounding variable family migration background as independent variables. Child age was included in both models to improve precision of the effect estimate after ensuring that it was not a collider, i.e., a common consequence of exposure and outcome [42]. To account for clustered data, random intercepts for schools, districts and time point were included. The model assumptions were checked graphically (S3 and S4 Figs). Regression coefficients and 95% confidence intervals were calculated, and the effects were interpreted according to Cohen’s *d* effect size measure for small (0.2), medium (0.5), and large (0.8) effects [42].

## Results

Overall, 660 students were enrolled in the study, of whom 480 (72.7%) participated at T1 and 377 (57.1%) at T3. Some children only responded once at T1 or T3, even though all participants were formally included at the same time point just before T1. A table describing the characteristics of non-respondents at one or both time points for comparison can be found in the supplement (see S2 Table). In total, 335 participants (50.8%) filled in the questionnaire at both time points and were weighted using IPW. The up-weighting ranged from 1.8 to 2.3, with a mean of 1.972. The resulting pseudo-population for both time points contained 660 students (see Table 1), equaling the original sample size. The pseudo-population consisted of 50.9% females and 49.1% males. At T1, the mean age was 12.9 years (SD = 2.3), and the age range was 6.8–19.1 years.

**Table 1:** Characteristics of the inverse probability weighted population at two time points.

	T1 (N = 660)	T3 (N = 660)
**Sex**		
female	336 (50.9%)	
male	325 (49.1%)	
**Age**		
mean (SD)	12.85 (2.27)	13.12 (2.27)
range	6.79–19.10	7.06–19.36
**School type** ^a^		
primary	241 (36.4%)	
secondary	420 (63.6%)	
**Age category**		
younger	238 (36.1%)	230 (34.9%)
older	422 (63.9%)	430 (65.1%)
**School district SES**		
high	393 (59.5%)	
middle	157 (23.7%)	
low	111 (16.8%)	
**Monthly net household income**		
higher	330 (51.7%)	343 (53.7%)
lower	308 (48.3%)	295 (46.3%)
missing	23	23
**Household education**		
higher	571 (86.8%)	573 (87.1%)
lower	87 (13.2%)	85 (12.9%)
missing	2	2
**Household size**		
larger	491 (74.4%)	469 (71.0%)
smaller	169 (25.6%)	192 (29.0%)
**Family migration background**		
no	514 (78.0%)	
yes	145 (22.0%)	
missing	2	

^a^In Berlin schools, the age range is 6–14 years for primary and 10–19 years for secondary schools.

*Note*. Due to the weight estimation process underlying the IPW approach, the non-integer participant weights sum up to 660.552, which is minimally above the original sample of 660. Because rounding would yield a sample of 661, we chose to report the integer part of 660 for the pseudo-population. Therefore, the sum of individual strata might be ±1 off from the total number of participants.

Most children and adolescents had no family migration background (78%). Households with a higher level of education (university entrance qualification or university degree) represented 86.8%. The monthly net household income was more than 5000€ in 51.7% of households. Most households counted four or more members (74.4%), and most children and adolescents went to school in a city district with high SES (59.5%).

**Fig 1:**
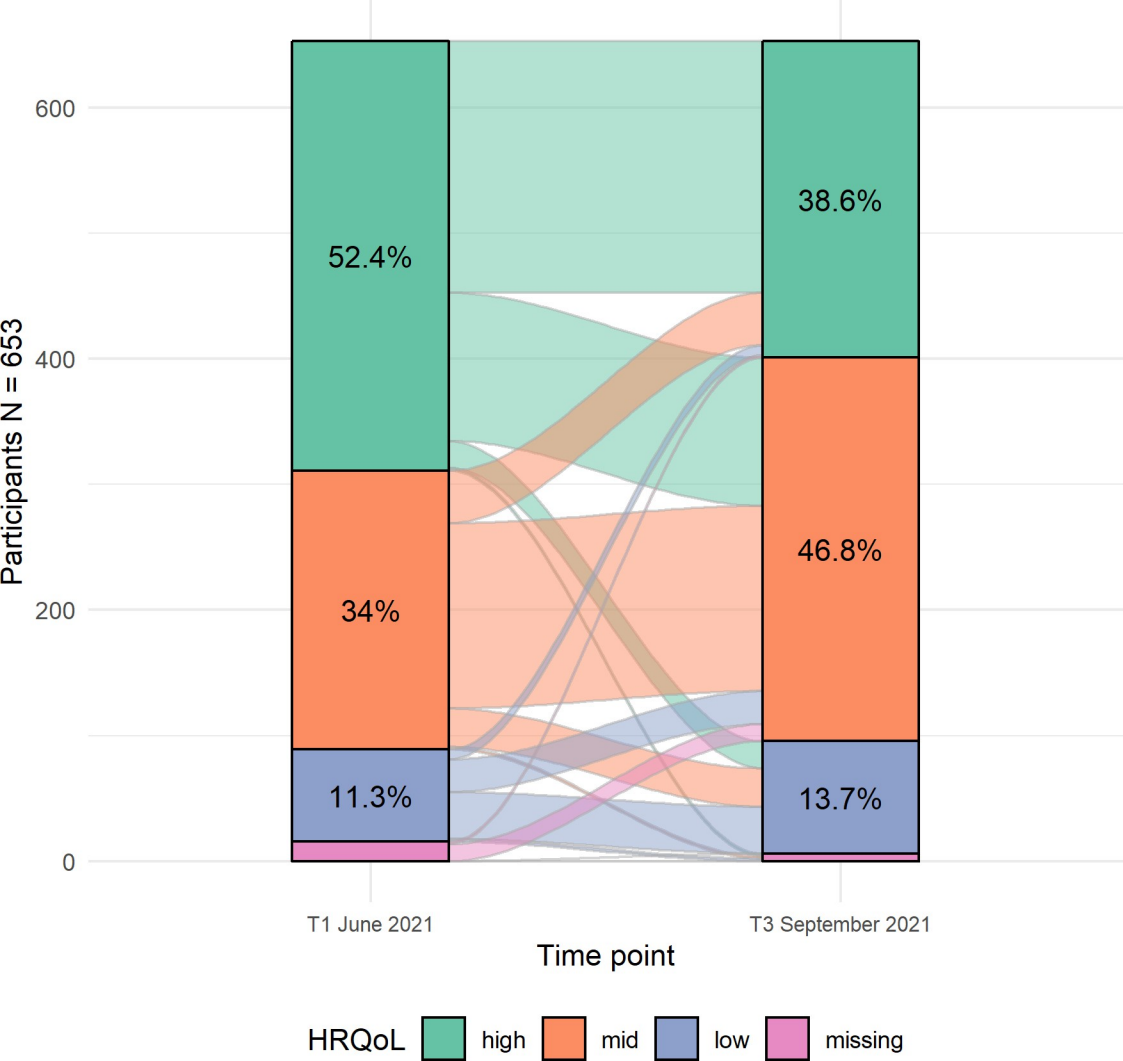
Individual changes in HRQoL categories across both time points for children aged 8–18 in the pseudo-population.

Table 2 depicts the HRQoL in terms of mean T-values, and Fig 1 shows the change between the HRQoL categories. Low HRQoL was reported by 11.3% [95% CI = 9.0% - 14.0%] of participants at T1 and 13.7% [95% CI = 11.1% - 16.5%] at T3. Changes in individuals’ HRQoL scores between the two time points occurred across all categories. Most participants (59% [95% CI = 55.1% - 62.8%]) remained in the same category at T1 and T3, but nearly 30% [95% CI = 26.5% - 33.7%] of children changed to a lower one at T3 (Fig 1).

**Table 2:** KIDSCREEN-10 health-related quality of life T-values for children aged 8–18 years in the pseudo-population.

	T1 (N = 653^a^)	T3 (N = 653^a^)
**HRQoL T-values**		
mean [95% CI]	55.34 [54.65–56.03]	53.11 [52.39–53.83]
range	29.97–83.81	32.87–83.81
missing	16	6

*Note*. Study participants who returned their questionnaire and had more than one missing item in the KIDSCREEN-10 were still weighted as part of the pseudo-population to stand in for non-responders and appear as “missing”

^a^Descriptive analyses were performed including children aged 8–18 years, which resulted in a slightly reduced pseudo-population of 653 students for both time points.

**Fig 2:**
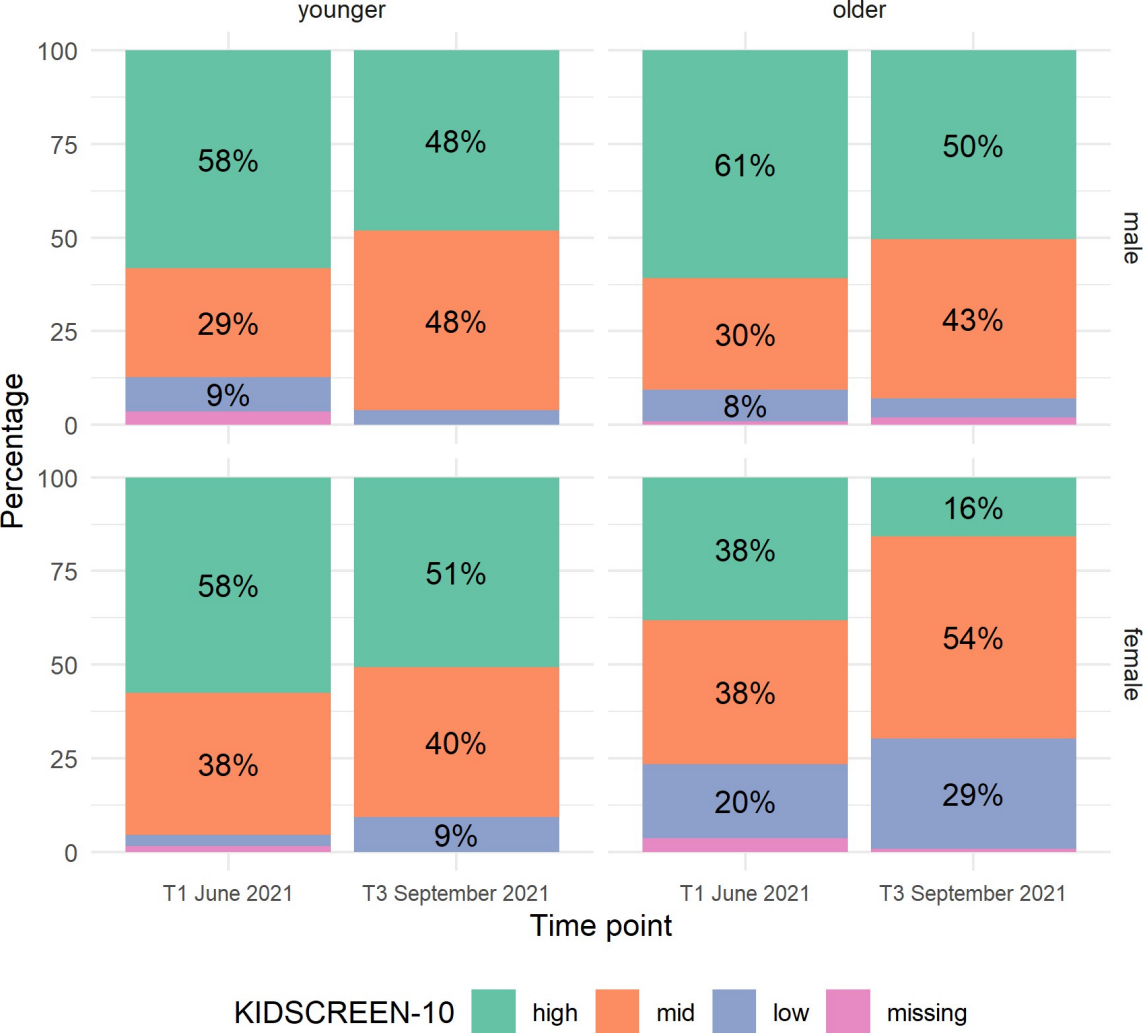
HRQoL categories by time point, sex, and age category (younger = 8 – 11 vs. older = 12 – 18 years).

Stratified by sex and age categories, sex differences in HRQoL were more pronounced within the older age stratum (see Fig 2). While less than 10% [95% CI = 8.7% - 12.5%] of boys of both age groups, as well as of girls younger than 12 years, reported low HRQoL at both time points, 20% [95% CI = 17.1% - 23.3%] of older girls reported low HRQoL at T1 and 29% [95% CI = 25.5% - 32.5%] at T3. In addition, we analyzed HRQoL categories by sex and school type to account for differences between primary and secondary schools regardless of age. The results differed only minimally (S5 Fig.).

We found no statistically significant effect of lower household income on HRQoL (*β* = -1.02, SE = 0.64, 95% CI = -2.28 to 0.23, *p* = 0.110). Interpreting the effect size in terms of Cohen’s *d* [4, 26, 39], the statistically non-significant negative effect of household income with a value of *d* = -0.11 can be considered very small. However, there was a statistically significant effect of lower household education on HRQoL (*β* = -2.15, SE = 0.95, 95% CI = -4.01 to -0.29, *p* = 0.024), indicating that lower household education (primary or secondary school levels) coincides with lower T-values of HRQoL by on average 2.15 units compared to higher household education (university entrance qualification or university degree). The statistically significant negative effect of household education with Cohen’s *d* = -0.23 represents a small effect.

## Discussion

Our study aimed to describe the HRQoL of primary and secondary school students in Berlin, Germany, at two time points directly before and six weeks after the summer vacation in 2021. After living approximately one and a half years with pandemic-related restrictions in school routine and social life, the studied cohort partly reported similar levels of HRQoL as in pre-pandemic times [4, 26, 43]. However, HRQoL differed across time points as well as strata of sex and age, with older girls more frequently reporting low HRQoL compared to boys of both age groups and to younger girls. This observation with respect to age and sex is in line with other studies [40] and has been reported prior to the COVID-19 pandemic [44]. Hypotheses about the mechanisms behind these differences include complex interactions of socio-behavioral and societal factors [43, 45] and must be investigated further [26].

The mean HRQoL T-values reported at both time points are mostly within the half a standard deviation range around the mean of the pre-pandemic European reference score. Only 11.3% of students reported low levels of HRQoL in June 2021 and 13.7% in September 2021, suggesting that low HRQoL was not generally elevated in the later pandemic stage in our cohort. Low levels and mean T-values of HRQoL in our sample were comparable to the results of the BELLA study in a pre-pandemic representative German sample [4, 24] and a German study similar to ours conducted in the same period [27]. However, other studies reported low HRQoL in between 35.5% to 48.1% of children and adolescents across the various stages of the pandemic [39], including one time point in early 2022. Kästner et al. hypothesized that intensified restricted measures in their study region over the winter 2021/22 before data collection were a possible explanation for elevated low HRQoL in their sample [43].

Our study took place during summer 2021 when pandemic-related restrictions were loosened and schools mostly open. Students in the study reported higher levels of HRQoL compared with results from other studies at earlier pandemic time points [4, 26, 46], which could be attributed to the easing of restrictions having a positive impact [47]. In June 2021 (T1), Berlin school students generally resumed classes with regular class sizes. However, school attendance was not yet mandatory. At T3 in September 2021, Berlin students had returned to regular school routine and compulsory attendance for six weeks already, mirroring a state of “new normalcy”.

Regarding effects of socioeconomic factors on students’ HRQoL following the third COVID-19 wave, we did not find support of a causal effect of lower (vs. higher) income on HRQoL. More than half of households reported a monthly net income of more than 5000€ and higher education levels. The low sample sizes in the lower income categories possibly did not provide enough statistical power to detect a potential effect of household income on the outcome. Nevertheless, we found that lower education reduced HRQoL of children by 2.1 T-value points compared to households with higher education. While this small effect does not automatically indicate a clinically relevant shift in HRQoL category, this finding potentially underscores the need to identify prevention target groups among children from households with lower education levels. Previous work has shown that parents may face challenges to compensate for their children’s learning losses following school closures during the pandemic [46], potentially increasing the risk of parental stress, which can have a negative impact on the wellbeing of their children in the longer term [47–49]. This risk may be higher if parents work in precarious jobs with high workloads or shift work, or lack the option to work from home, which is common in professions with lower educational levels [46]. Nevertheless, our results cannot be interpreted as a consequence of the pandemic as we are lacking a pre-pandemic time point and may stem from both pre-existing inequalities and an exacerbation during the pandemic.

Our findings contrast with those of a study conducted in several European countries before the COVID-19 pandemic, which indicated that family wealth had a stronger impact on child and adolescent HRQoL than parental education [50]. Instead of household income, indicators of family wealth were operationalized using the family affluence scale [20, 47, 48] including questions that were child-specific like having one’s own bedroom. It is possible that household income does not adequately assess material resources to examine the impact on HRQoL of children and adolescents, and other, more relevant indicators such as housing or access to technology should be included in future studies, depending on the study setting.

Studies often evaluate SES as a composite score [20, 51, 52], although the single components of SES may affect the outcome through different pathways, and the discovered effects may differ [50, 51], as shown in this study. Further research is needed that investigates socioeconomic influencing factors on the different dimensions of students’ HRQoL during the pandemic separately, as this may provide further insight into the exact mechanisms at play.

### Strengths and limitations

The present study assessed HRQoL in children and adolescents at a later COVID-19 pandemic stage in the summer of 2021 in Berlin, Germany. The data from two time points were combined for assessment of the causal research questions to obtain a more accurate estimate that is less impacted by pandemic-related variations. Other strengths are the use of an internationally applied and validated instrument (KIDSCREEN-10 self-report Index), IPW to partially account for non-response bias and non-random attrition based on age, sex, and participation at baseline, and DAGs to formalize the causal framework underlying our research questions. Moreover, the study included the perspective of children and adolescents themselves.

This study has several limitations. We decided to use the shortened version of the KIDSCREEN instrument instead of the KIDSCREEN-52, as the comprehensive questionnaire captures a wide range of variables and reliability measures are comparable (Cronbach’s alpha of 0.82 for KIDSCREEN-10) [53]. Our sample is unbalanced, containing more households with higher income and education. In addition, there might be selection bias concerning differing student populations in the participating schools compared to those that rejected participation, and within schools in selecting participating classes. Our sample is therefore most likely not representative of the school student population in Berlin, Germany. Specifically, only 22% of participants in our sample reported a family migration background, while more than 51% of children aged between 6 and 18 years had a migration background in Berlin in 2021 [54, 55]. Due to the study’s limits in representativeness and specific setting, generalizability of the results is limited. Migration background, socioeconomic factors, and lower HRQoL might affect non-response and non-random attrition which could not be accounted for in IPW due to missingness in some of these variables. Therefore, and also since a small effect of lower household education was shown, the prevalence of low HRQoL could be underestimated in our study due to an overrepresentation of higher SES households. Potential barriers to participation in our study include competing priorities and demands of daily life, lack of trust in research, fear of stigma, and lack of time or interest [54, 55]. Further, we asked parents of primary school students to help their children fill in the questionnaire, which may have influenced the children’s responses.

It is not possible to allocate the results of this study as direct causes of the pandemic itself or pandemic responses that may have resulted in job losses and income reduction. Because the time points in this study were within three months of each other while the pandemic was ongoing, possible changes in income due to pandemic-related circumstances cannot be determined.

Due to our study design, our causal analyses should be interpreted with caution. Several other individual and societal factors may have influenced the students’ HRQoL, some of which were not measured like single parenting, religious affiliation, parents’ professions, and number of adults and children in the household to calculate an equalized income measure adjusted for different household compositions. Nonetheless, we refrain from fully avoiding causal language, as this may lead to the adoption of ambiguous terms and could inadvertently obscure the clarity of study objectives and the plausibility of causal assumptions, as pointed out by Haber et al. [56]. Our paper strives for transparency in clearly articulating our assumptions.

## Conclusions

This study describes the HRQoL in school students on two occasions in June and September 2021 in Berlin, Germany, assessing the separate effects of household income and education on the children’s and adolescents’ HRQoL. Overall, the participants reported higher levels of HRQoL compared with other studies mostly from earlier pandemic time points, possibly reflecting the positive impact of loosened pandemic-related restrictions.

While household income showed no effect on HRQoL in the present sample, our results suggest that children from households with lower levels of education are at significant risk for reduced HRQoL, even though the effect is small. Support strategies for psychosocial wellbeing should consider socioeconomically disadvantaged children as important target groups. We suggest further in-depth subgroup analyses and research on pathways for the separate effects of socioeconomic variables on pandemic outcomes of psychosocial health and wellbeing among schoolchildren.

## Supporting information

S1 Checklist STROBE statement—checklist of items that should be included in reports of observational studies. (DOCX)

S1 File Sensitivity analysis with IPW including city district. Linear mixed model 1 for the total causal effect of household income on HRQoL for pseudo-population including city district in IPW.
(PDF)

S2 File Sensitivity analysis with IPW including city district. Linear mixed model 2 for the total causal effect of household education on HRQoL for pseudo-population including city district in IPW.
(PDF)

S1 Table Sensitivity analysis with IPW including city district. Characteristics of pseudo-population including city district in IPW.
(PDF)

S2 Table Comparing non-participants to participants. (PDF)

S1 Fig DAG for the total causal effect of household income on HRQoL. (PDF)

S2 Fig DAG for the total causal effect of household education on HRQoL. (PDF)

S3 Fig Performance of model 1 for the total causal effect of household income on HRQoL. (PDF)

S4 Fig Performance of model 2 for the total causal effect of household education on HRQoL. (PDF)

S5 Fig HRQoL categories by time point, sex, and school type (primary vs. secondary) based on the IPW pseudo-population of 653 children. (PDF)
