# Supplementary material for: Health-related quality of life and impact of socioeconomic status among primary and secondary school students after the third COVID-19 wave in Berlin, Germany
Source: PLoS One. 2024 May 9;19(5):e0302995. doi: 10.1371/journal.pone.0302995 (PMC11081372; doi:10.1371/journal.pone.0302995)
Supplement: S1 Checklist — (DOCX) [file pone.0302995.s001.docx]

STROBE Statement—checklist of items that should be included in reports of observational studies

|  | Item No. | Recommendation | Page  No. | Relevant text from manuscript |
| --- | --- | --- | --- | --- |
| **Title and abstract** | 1 | (*a*) Indicate the study’s design with a commonly used term in the title or the abstract | 1 | “In this cohort study…” |
|  |  | (*b*) Provide in the abstract an informative and balanced summary of what was done and what was found | 1/2 |  |
| Introduction | | | |  |
| Background/rationale | 2 | Explain the scientific background and rationale for the investigation being reported | 2/3 |  |
| Objectives | 3 | State specific objectives, including any prespecified hypotheses | 4 | “As a part of the longitudinal cohort study BECOSS 2 [28], the present study aimed at (1) describing the distribution of HRQoL in students on two occasions in June and September 2021 and (2) identifying socioeconomic determinants of HRQoL within the cohort. We hypothesized that lower household income and lower household education had a negative effect on HRQoL of schoolchildren.” |
| Methods | | | |  |
| Study design | 4 | Present key elements of study design early in the paper | 4/5 |  |
| Setting | 5 | Describe the setting, locations, and relevant dates, including periods of recruitment, exposure, follow-up, and data collection | 4/5 |  |
| Participants | 6 | (*a*) *Cohort study*—Give the eligibility criteria, and the sources and methods of selection of participants. Describe methods of follow-up  *Case-control study*—Give the eligibility criteria, and the sources and methods of case ascertainment and control selection. Give the rationale for the choice of cases and controls  *Cross-sectional study*—Give the eligibility criteria, and the sources and methods of selection of participants | 4/5 | “For random school selection, the twelve Berlin city districts were first divided into three socioeconomic strata, “low”, “middle”, and “high” [30]. Within each stratum, two districts were randomly selected. All public schools from the selected districts were then put in random order and approached in that sequence until two primary and two secondary schools from each SES stratum agreed to participate [23]. The resulting 24 schools chose participating classes depending on factors like teachers’ willingness and disposability regarding exams or school trips during the study period.  As described elsewhere [28], study information and consent forms were sent to the participating schools and distributed to the selected classes before the start of data collection. After the children and their guardians consented to study participation, paper-based questionnaires with instructions for completion were mailed to the home address of the enrolled participants at each study time point. Parents were asked to answer questions on the socioeconomic status of the household in a designated section on the first page of the questionnaire. For the participants in primary schools, parents were also encouraged to help their children with the questionnaire.” |
|  |  | (*b*) *Cohort study*—For matched studies, give matching criteria and number of exposed and unexposed  *Case-control study*—For matched studies, give matching criteria and the number of controls per case |  | Not matched |
| Variables | 7 | Clearly define all outcomes, exposures, predictors, potential confounders, and effect modifiers. Give diagnostic criteria, if applicable | 5-8 | See “Measures” and “Data analysis” |
| Data sources/ measurement | 8* | For each variable of interest, give sources of data and details of methods of assessment (measurement). Describe comparability of assessment methods if there is more than one group | 5/6 | See “Measures” |
| Bias | 9 | Describe any efforts to address potential sources of bias | 7/8 | “To account for non-response bias and non-random attrition, inverse probability weighting (IPW) was used. Participants were inversely weighted separately at each of the two time points based on their age, sex, and for T3 by participation at T1. These weights were then multiplied across both time points to produce an IPW‑population, referred to as “pseudo‑population”. All analyses were conducted within the pseudo-population, based on the weighted responses of children and adolescents with complete observations in T1 and T3 to account for missing data from non-respondents of the same age and sex.”; … “Variable selection and construction of the causal diagrams to establish potential confounding and mediating variables were based on subject matter knowledge and previous research on causal relationships of SES (or income and education separately) and health outcomes [5,9,17,20,35–38]. Minimal adjustment sets for variables potentially leading to confounding according to the causal diagram were determined using the software DAGitty [39].” |
| Study size | 10 | Explain how the study size was arrived at | 8 | “Overall, 660 students were enrolled in the study, of whom 480 (72.7%) participated at T1 and 377 (57.1%) at T3. Using IPW, the resulting pseudo-population for both time points contained 660 students (see Table 1), equaling the original sample size.” |

Continued on next page

| Quantitative variables | 11 | Explain how quantitative variables were handled in the analyses. If applicable, describe which groupings were chosen and why | 7/8 |  |
| --- | --- | --- | --- | --- |
| Statistical methods | 12 | (*a*) Describe all statistical methods, including those used to control for confounding | 7/8 | “For the analysis of the causal research questions, assumptions on the underlying causal relations between the two exposures (income, education) and the outcome (HRQoL) with all potentially relevant variables from the BECOSS 2 dataset were depicted graphically using directed acyclic graphs (DAGs) separately for each effect [33,34] (see Additional file 1: Figs S1 and S2). Variable selection and construction of the causal diagrams to establish potential confounding and mediating variables were based on subject matter knowledge and previous research on causal relationships of SES (or income and education separately) and health outcomes [5,9,17,20,35–38]. Minimal adjustment sets for variables potentially leading to confounding according to the causal diagram were determined using the software DAGitty [39].  Two generalized linear mixed models were set up to estimate the total effects of household income and education on HRQoL separately. The first model used T-values of HRQoL as the dependent variable and household income as well as the potential confounding variables household size, education, and family migration background as independent variables. The second model used T-values of HRQoL as the dependent variable and household education plus the potential confounding variable family migration background as independent variables. Child age was included in both models to improve precision of the effect estimate after ensuring that it was not a collider, i.e., a common consequence of exposure and outcome [33]. To account for clustered data, random intercepts for schools, districts and time point were included. The model assumptions were checked graphically (see Additional file 1: Figs S3 and S4). Regression coefficients and 95% confidence intervals were calculated, and the effects were interpreted according to Cohen’s d effect size measure for small (0.2), medium (0.5), and large (0.8) effects [40].” |
|  |  | (*b*) Describe any methods used to examine subgroups and interactions | 7/8 | See above |
|  |  | (*c*) Explain how missing data were addressed | 7/8 | “To account for non-response bias and non-random attrition, inverse probability weighting (IPW) was used. Participants were inversely weighted separately at each of the two time points based on their age, sex, and for T3 by participation at T1. These weights were then multiplied across both time points to produce an IPW population, referred to as “pseudo population”. All analyses were conducted within the pseudo-population, based on the weighted responses of children and adolescents with complete observations in T1 and T3 to account for missing data from non-respondents of the same age and sex.” |
|  |  | (*d*) *Cohort study*—If applicable, explain how loss to follow-up was addressed  *Case-control study*—If applicable, explain how matching of cases and controls was addressed  *Cross-sectional study*—If applicable, describe analytical methods taking account of sampling strategy |  | See above |
|  |  | (*e*) Describe any sensitivity analyses |  | See above |
| Results | | | | |
| Participants | 13* | (a) Report numbers of individuals at each stage of study—eg numbers potentially eligible, examined for eligibility, confirmed eligible, included in the study, completing follow-up, and analysed | 8 |  |
|  |  | (b) Give reasons for non-participation at each stage | 13 | Limitations: “Potential barriers to participation in our study include competing priorities and demands of daily life, lack of trust in research, fear of stigma, and lack of time or interest [51,52]” |
|  |  | (c) Consider use of a flow diagram |  |  |
| Descriptive data | 14* | (a) Give characteristics of study participants (eg demographic, clinical, social) and information on exposures and potential confounders | 9 | Table 1 |
|  |  | (b) Indicate number of participants with missing data for each variable of interest |  | Not applicable (due to IPW) |
|  |  | (c) *Cohort study*—Summarise follow-up time (eg, average and total amount) | 8 |  |
| Outcome data | 15* | *Cohort study*—Report numbers of outcome events or summary measures over time | 10 |  |
|  |  | *Case-control study—*Report numbers in each exposure category, or summary measures of exposure | - |  |
|  |  | *Cross-sectional study—*Report numbers of outcome events or summary measures | - |  |
| Main results | 16 | (*a*) Give unadjusted estimates and, if applicable, confounder-adjusted estimates and their precision (eg, 95% confidence interval). Make clear which confounders were adjusted for and why they were included | 10 | See Results and Figures |
|  |  | (*b*) Report category boundaries when continuous variables were categorized |  |  |
|  |  | (*c*) If relevant, consider translating estimates of relative risk into absolute risk for a meaningful time period |  |  |

Continued on next page

| Other analyses | 17 | Report other analyses done—eg analyses of subgroups and interactions, and sensitivity analyses |  |  |
| --- | --- | --- | --- | --- |
| Discussion | | | | |
| Key results | 18 | Summarise key results with reference to study objectives | 11 | “Our study aimed to describe the HRQoL of primary and secondary school students in Berlin, Germany, at two time points directly before and six weeks after the summer vacation in 2021. After living approximately one and a half years with pandemic-related restrictions in school routine and social life, the studied cohort partly reported similar levels of HRQoL as in pre-pandemic times [4,22]. However, HRQoL differed across time points as well as strata of sex and age, with older girls more frequently reporting low HRQoL compared to boys of both age groups and to younger girls. This observation with respect to age and sex is in line with other studies [4,26,41] and has been reported prior to the COVID-19 pandemic [22]. Hypotheses about the causal mechanisms behind these differences include complex interactions of socio-behavioral and societal factors [42] and must be investigated further [41,43].” |
| Limitations | 19 | Discuss limitations of the study, taking into account sources of potential bias or imprecision. Discuss both direction and magnitude of any potential bias | 13 | “This study has several limitations. Our sample is unbalanced, containing more households with higher income and education. In addition, there might be selection bias concerning differing student populations in the participating schools compared to those that rejected participation, and within schools in selecting participating classes. Our sample is therefore most likely not representative of the school student population in Berlin, Germany. Potential barriers to participation in our study include competing priorities and demands of daily life, lack of trust in research, fear of stigma, and lack of time or interest [51,52]. Further, we asked parents of primary school students to help their children fill in the questionnaire, which may have influenced the children’s responses.  Finally, it is not possible to allocate the results of this study as direct causes of the pandemic itself or pandemic responses that may have resulted in job losses and income reduction. Because the time points in this study were within three months of each other while the pandemic was ongoing, possible changes in income due to pandemic-related circumstances cannot be determined. Several other individual and societal factors may have influenced the students’ HRQoL, some of which were not measured like single parenting, religious affiliation, parents’ professions, and number of adults and children in the household to calculate an equalized income measure adjusted for different household compositions.” |
| Interpretation | 20 | Give a cautious overall interpretation of results considering objectives, limitations, multiplicity of analyses, results from similar studies, and other relevant evidence | 14 | “This study describes the HRQoL in school students on two occasions in June and September 2021 in Berlin, Germany, assessing the separate effects of household income and education on the children’s and adolescents’ HRQoL. Overall, the participants reported higher levels of HRQoL compared with other studies mostly from earlier pandemic time points, possibly reflecting the positive impact of loosened pandemic-related restrictions.  While household income showed no effect on HRQoL in the present sample, our results suggest that children from households with lower levels of education are at significant risk for reduced HRQoL, even though the effect is small. Support strategies for psychosocial wellbeing should consider socioeconomically disadvantaged children as important target groups. We suggest further in-depth subgroup analyses and research on pathways for the separate effects of socioeconomic variables on pandemic outcomes of psychosocial health and wellbeing among schoolchildren.” |
| Generalisability | 21 | Discuss the generalisability (external validity) of the study results | 13 | “In addition, there might be selection bias concerning differing student populations in the participating schools compared to those that rejected participation, and within schools in selecting participating classes. Our sample is therefore most likely not representative of the school student population in Berlin, Germany.” |
| Other information | |  | | |
| Funding | 22 | Give the source of funding and the role of the funders for the present study and, if applicable, for the original study on which the present article is based |  | The study was funded by the Senate of Berlin. The donor had no role in study design, data collection and analysis decision to publish and preparation of the manuscript. |

*Give information separately for cases and controls in case-control studies and, if applicable, for exposed and unexposed groups in cohort and cross-sectional studies.

**Note:** An Explanation and Elaboration article discusses each checklist item and gives methodological background and published examples of transparent reporting. The STROBE checklist is best used in conjunction with this article (freely available on the Web sites of PLoS Medicine at http://www.plosmedicine.org/, Annals of Internal Medicine at http://www.annals.org/, and Epidemiology at http://www.epidem.com/). Information on the STROBE Initiative is available at www.strobe-statement.org.
