# Supplementary material for: Health-related quality of life and impact of socioeconomic status among primary and secondary school students after the third COVID-19 wave in Berlin, Germany
Source: PLoS One. 2024 May 9;19(5):e0302995. doi: 10.1371/journal.pone.0302995 (PMC11081372; doi:10.1371/journal.pone.0302995)
Supplement: S1 File — Linear mixed model 1 for the total causal effect of household income on HRQoL for pseudo-population including city district in IPW. (PDF) [file pone.0302995.s002.pdf]

S1 Results Model 1. Sensitivity analysis with IPW including city district. Linear mixed model 1 for the total causal effect of household income on HRQoL for pseudo-population including city district in IPW.

```
## Linear mixed model fit by REML. t-tests use Satterthwaite's method ['lmerModLmerTest']
## Formula: T_score_selfReportEU ~ Household_income + Household_size + Family_migration_backgr
ound +      Household_education + Alter + (1 | wave) + (1 | IDSchule) +      (1 | Bezirk)
##    Data: dat_district_lm
## Weights: weight13MM
##
## REML criterion at convergence: Inf
##
## Scaled residuals:
##     Min      1Q  Median      3Q     Max
## -2.9615 -0.5842  0.0000  0.3559  4.3609
##
## Random effects:
##  Groups   Name        Variance Std.Dev.
##  IDSchule (Intercept)  13.3196  3.6496
##  Bezirk   (Intercept)   4.3559  2.0871
##  wave     (Intercept)   0.7146  0.8453
##  Residual             105.4115 10.2670
## Number of obs: 800, groups:  IDSchule, 23; Bezirk, 6; wave, 2
##
## Fixed effects:
##                                Estimate Std. Error       df t value Pr(>|t|)
## (Intercept)                     72.1321     3.0961  12.3445  23.297 1.41e-11 ***
## Household_incomelower           -0.9693     0.6414 788.4900  -1.511   0.1311
## Household_sizesmaller            0.4280     0.6981 785.2578   0.613   0.5400
## Family_migration_backgroundyes  -0.4353     0.7739 775.0845  -0.562   0.5740
## Household_educationlower        -1.8204     0.9744 717.1828  -1.868   0.0621 .
## Alter                           -1.3638     0.2260  69.5432  -6.036 6.90e-08 ***
## ---
## Signif. codes:  0 '***' 0.001 '**' 0.01 '*' 0.05 '.' 0.1 ' ' 1
##
## Correlation of Fixed Effects:
##             (Intr) Hshld_n Hshld_s Fmly__ Hshld_d
## Hshld_ncmlw -0.033
## Hshld_szsml  0.015 -0.105
## Fmly_mgrtn_  0.018 -0.042   0.020
## Hshld_dctnl  0.139 -0.245  -0.071  -0.018
## Alter       -0.887 -0.064  -0.064  -0.080 -0.180
## optimizer (nloptwrap) convergence code: 0 (OK)
## Gradient contains NAs
```
