# Supplementary material for: Health-related quality of life and impact of socioeconomic status among primary and secondary school students after the third COVID-19 wave in Berlin, Germany
Source: PLoS One. 2024 May 9;19(5):e0302995. doi: 10.1371/journal.pone.0302995 (PMC11081372; doi:10.1371/journal.pone.0302995)
Supplement: S2 File — Linear mixed model 2 for the total causal effect of household education on HRQoL for pseudo-population including city district in IPW. (PDF) [file pone.0302995.s003.pdf]

S2 Results Model 2 **Sensitivity analysis with IPW including city district. Linear mixed model 2 for the total causal effect of household education on HRQoL for pseudo-population including city district in IPW.**

```
## Linear mixed model fit by REML. t-tests use Satterthwaite's method ['lmerModLmerTest']
## Formula: T_score_selfReportEU ~ Household_education + Family_migration_background +      Al
ter + (1 | wave) + (1 | IDSchule) + (1 | Bezirk)
##    Data: dat_district_lm
## Weights: weight13MM
##
## REML criterion at convergence: Inf
##
## Scaled residuals:
##     Min      1Q  Median      3Q     Max
## -3.0503 -0.5629  0.0000  0.3728  4.2830
##
## Random effects:
##  Groups   Name        Variance Std.Dev.
##  IDSchule (Intercept)  13.3279  3.6507
##  Bezirk   (Intercept)   4.3586  2.0877
##  wave     (Intercept)   0.7151  0.8456
##  Residual             105.4768 10.2702
## Number of obs: 800, groups:  IDSchule, 23; Bezirk, 6; wave, 2
##
## Fixed effects:
##                                Estimate Std. Error       df t value Pr(>|t|)
## (Intercept)                     71.9601     3.0952  14.8471  23.249 4.34e-13 ***
## Household_educationlower        -2.1384     0.9401 713.6116  -2.275   0.0232 *
## Family_migration_backgroundyes  -0.4903     0.7734 779.3595  -0.634   0.5263
## Alter                           -1.3784     0.2250  77.7555  -6.126 3.45e-08 ***
## ---
## Signif. codes:  0 '***' 0.001 '**' 0.01 '*' 0.05 '.' 0.1 ' ' 1
##
## Correlation of Fixed Effects:
##             (Intr) Hshld_ Fmly__
## Hshld_dctnl  0.137
## Fmly_mgrtn_  0.017 -0.028
## Alter       -0.893 -0.211 -0.082
## optimizer (nloptwrap) convergence code: 0 (OK)
## Gradient contains NAs
```
