# Supplementary material for: Health-related quality of life and impact of socioeconomic status among primary and secondary school students after the third COVID-19 wave in Berlin, Germany
Source: PLoS One. 2024 May 9;19(5):e0302995. doi: 10.1371/journal.pone.0302995 (PMC11081372; doi:10.1371/journal.pone.0302995)
Supplement: S1 Table — Characteristics of pseudo-population including city district in IPW. (PDF) [file pone.0302995.s004.pdf]

S3 Table Sensitivity Analysis with IPW including city district. Characteristics of pseudo-population including city district in IPW.

	T1 (N = 644)		T3 (N = 644)
**Sex**			
female		333 (51.8%)	
male		310 (48.2%)	
**Age**			
mean (SD)	12.772 (2.330)		13.040 (2.330)
range	6.79 – 19.10		7.06 – 19.36
**School type**[a]			
primary		249 (38.7%)	
secondary		394 (61.3%)	
**Age category**			
younger	246 (38.2%)		238 (37.0%)
older	398 (61.8%)		406 (63.0%)
**School district SES**			
high		335 (52.0%)	
middle		150 (23.3%)	
low		159 (24.7%)	
**Monthly net household income**			
higher	319 (49.5%)		327 (50.8%)
lower	304 (47.2%)		295 (45.9%)
missing	22		22
**Household education**			
higher	551 (85.5%)		552 (85.7%)
lower	91 (14.2%)		90 (14.0%)
missing	2		2
**Household size**			
larger	483 (75.1%)		461 (71.6%)
smaller	161 (24.9%)		183 (28.4%)
**Family migration background**			
no		496 (77.0%)	
yes		146 (22.7%)	
missing		2	

Note. Since the non-integer participant weights sum up to 643.7885755, the rounded total number of the IPW pseudo-population is 644.
