# Supplementary material for: Health-related quality of life and impact of socioeconomic status among primary and secondary school students after the third COVID-19 wave in Berlin, Germany
Source: PLoS One. 2024 May 9;19(5):e0302995. doi: 10.1371/journal.pone.0302995 (PMC11081372; doi:10.1371/journal.pone.0302995)
Supplement: S2 Table — (PDF) [file pone.0302995.s005.pdf]

S8 Table Comparing non-participants to participants.

	Non-Participants		Participants	
	T1 (N=180)	T3 (N=283)	T1 (N=335)	T3 (N=335)
**Sex**				
female	86 (47.8%)	137 (48.4%)	179 (53.4%)	179 (53.4%)
male	94 (52.2%)	146 (51.6%)	156 (46.6%)	156 (46.6%)
**Age**				
Mean (SD)	12.5 (2.47)	13.3 (2.32)	12.8 (2.27)	13.0 (2.27)
Median [Min, Max]	13.0 [6.98, 19.1]	13.6 [7.25, 19.4]	13.0 [6.79, 17.6]	13.3 [7.06, 17.9]
**School type**				
primary	75 (41.7%)	96 (33.9%)	126 (37.6%)	126 (37.6%)
secondary	105 (58.3%)	187 (66.1%)	209 (62.4%)	209 (62.4%)
**Age category**				
older	106 (58.9%)	193 (68.2%)	210 (62.7%)	214 (63.9%)
younger	74 (41.1%)	90 (31.8%)	125 (37.3%)	121 (36.1%)
**School district SES**				
high	63 (35.0%)	107 (37.8%)	198 (59.1%)	198 (59.1%)
middle	47 (26.1%)	65 (23.0%)	80 (23.9%)	80 (23.9%)
low	70 (38.9%)	111 (39.2%)	57 (17.0%)	57 (17.0%)
**Monthly net household income**				
higher	25 (13.9%)	57 (20.1%)	168 (50.1%)	175 (52.2%)
lower	42 (23.3%)	101 (35.7%)	156 (46.6%)	149 (44.5%)
missing	113 (62.8%)	125 (44.2%)	11 (3.3%)	11 (3.3%)
**Household education**				
higher	61 (33.9%)	126 (44.5%)	290 (86.6%)	291 (86.9%)
lower	13 (7.2%)	50 (17.7%)	44 (13.1%)	43 (12.8%)
missing	106 (58.9%)	107 (37.8%)	1 (0.3%)	1 (0.3%)
**Household size**				
larger	50 (27.8%)	126 (44.5%)	251 (74.9%)	240 (71.6%)
missing	106 (58.9%)	108 (38.2%)	0 (0%)	0 (0%)
smaller	24 (13.3%)	49 (17.3%)	84 (25.1%)	95 (28.4%)
**Family migration background**				
missing	106 (58.9%)	108 (38.2%)	1 (0.3%)	1 (0.3%)
no	50 (27.8%)	113 (39.9%)	261 (77.9%)	261 (77.9%)
yes	24 (13.3%)	62 (21.9%)	73 (21.8%)	73 (21.8%)
