# Supplementary figures and images for: Health-related quality of life and impact of socioeconomic status among primary and secondary school students after the third COVID-19 wave in Berlin, Germany
Source: PLoS One. 2024 May 9;19(5):e0302995. doi: 10.1371/journal.pone.0302995 (PMC11081372; doi:10.1371/journal.pone.0302995)

**S4 Fig. DAG for the total causal effect of household income on HRQoL.**

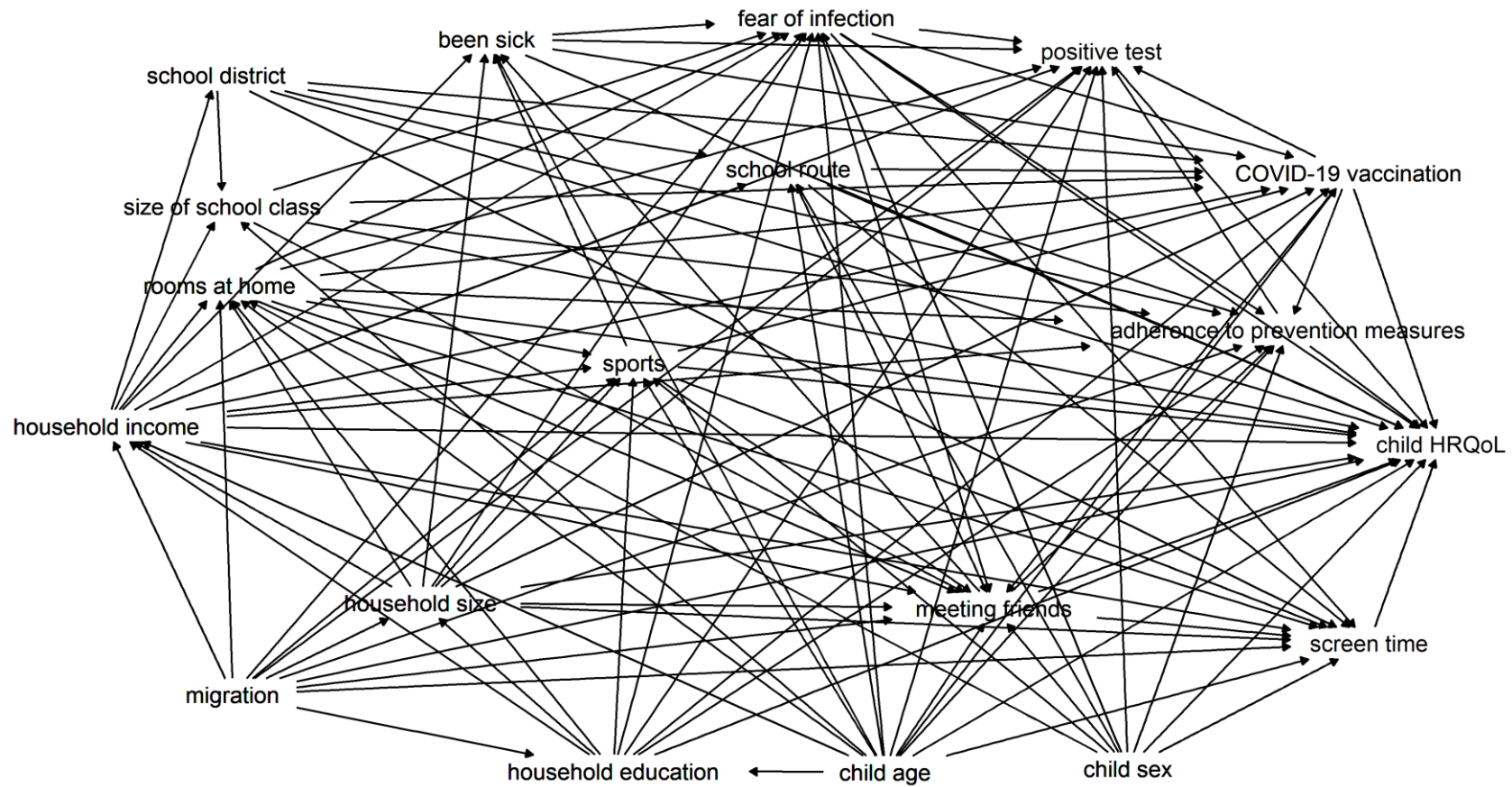

Supplement: S1 Fig — (PDF) [file pone.0302995.s006.pdf]

S5 Fig. DAG for the total causal effect of household education on HRQoL.

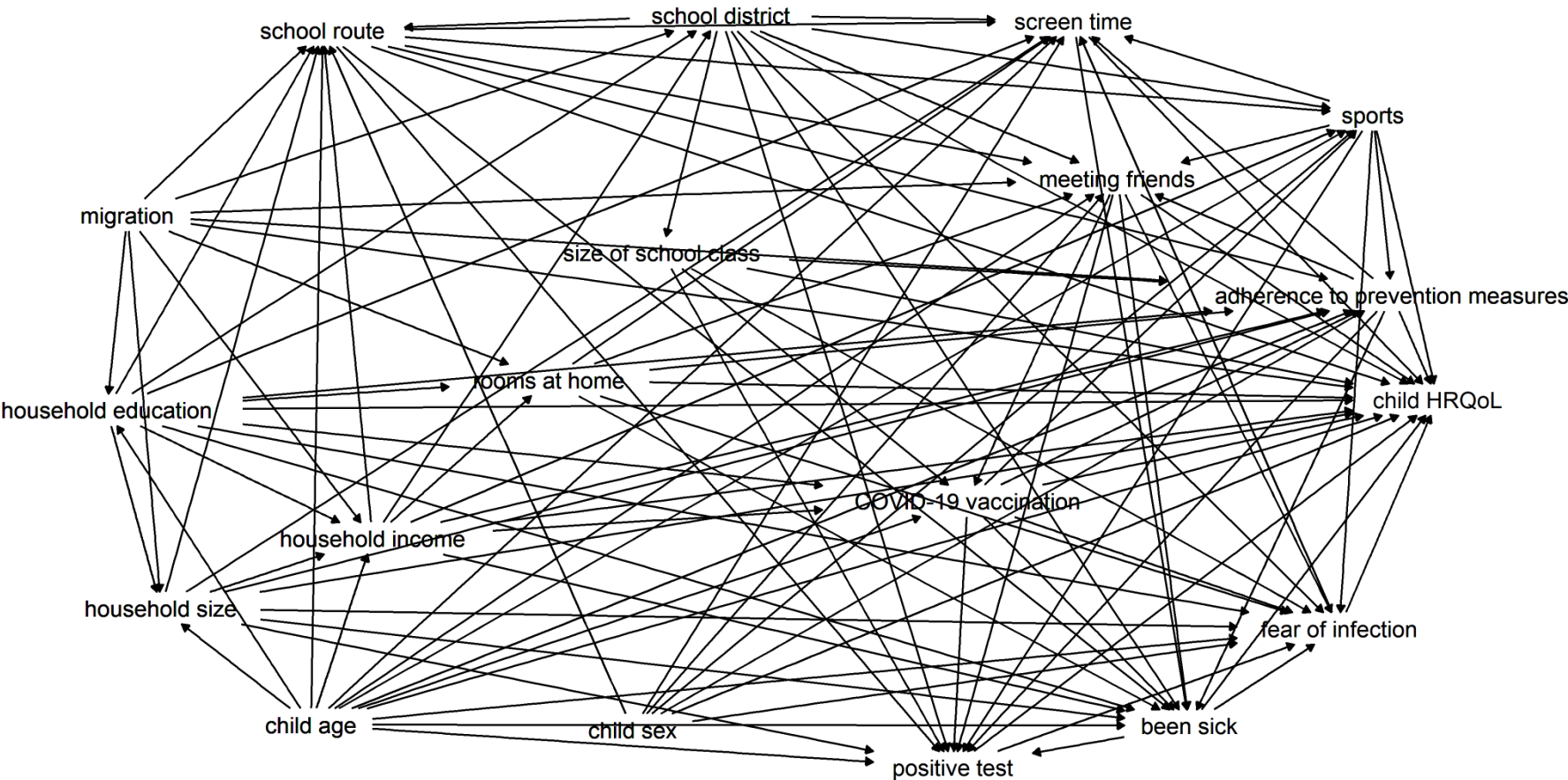

Supplement: S2 Fig — (PDF) [file pone.0302995.s007.pdf]

**S6 Fig. Performance of model 1 for the total causal effect of household income on HRQoL.**

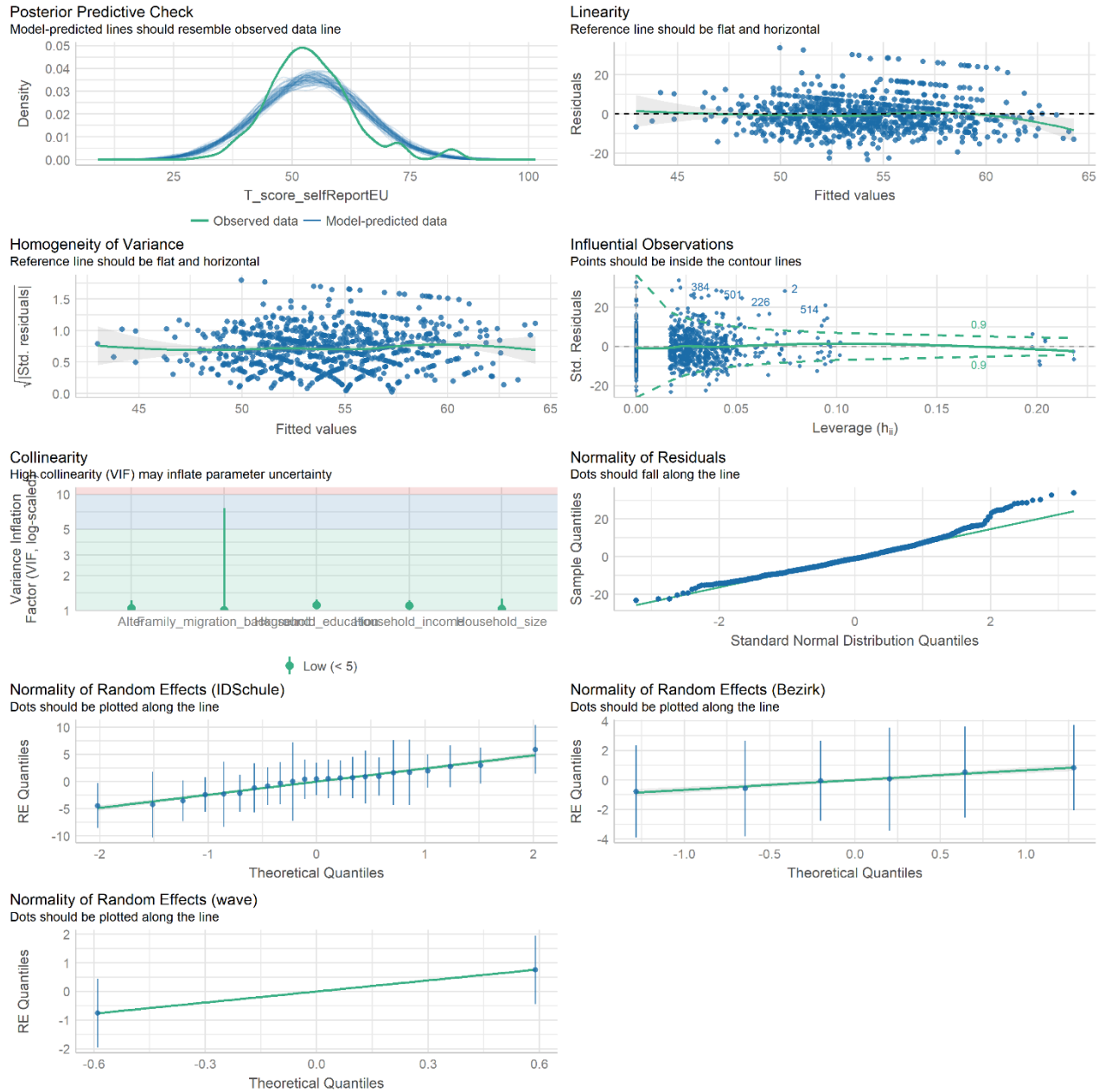

Supplement: S3 Fig — (PDF) [file pone.0302995.s008.pdf]

**S7 Fig. Performance of model 2 for the total causal effect of household education on HRQoL.**

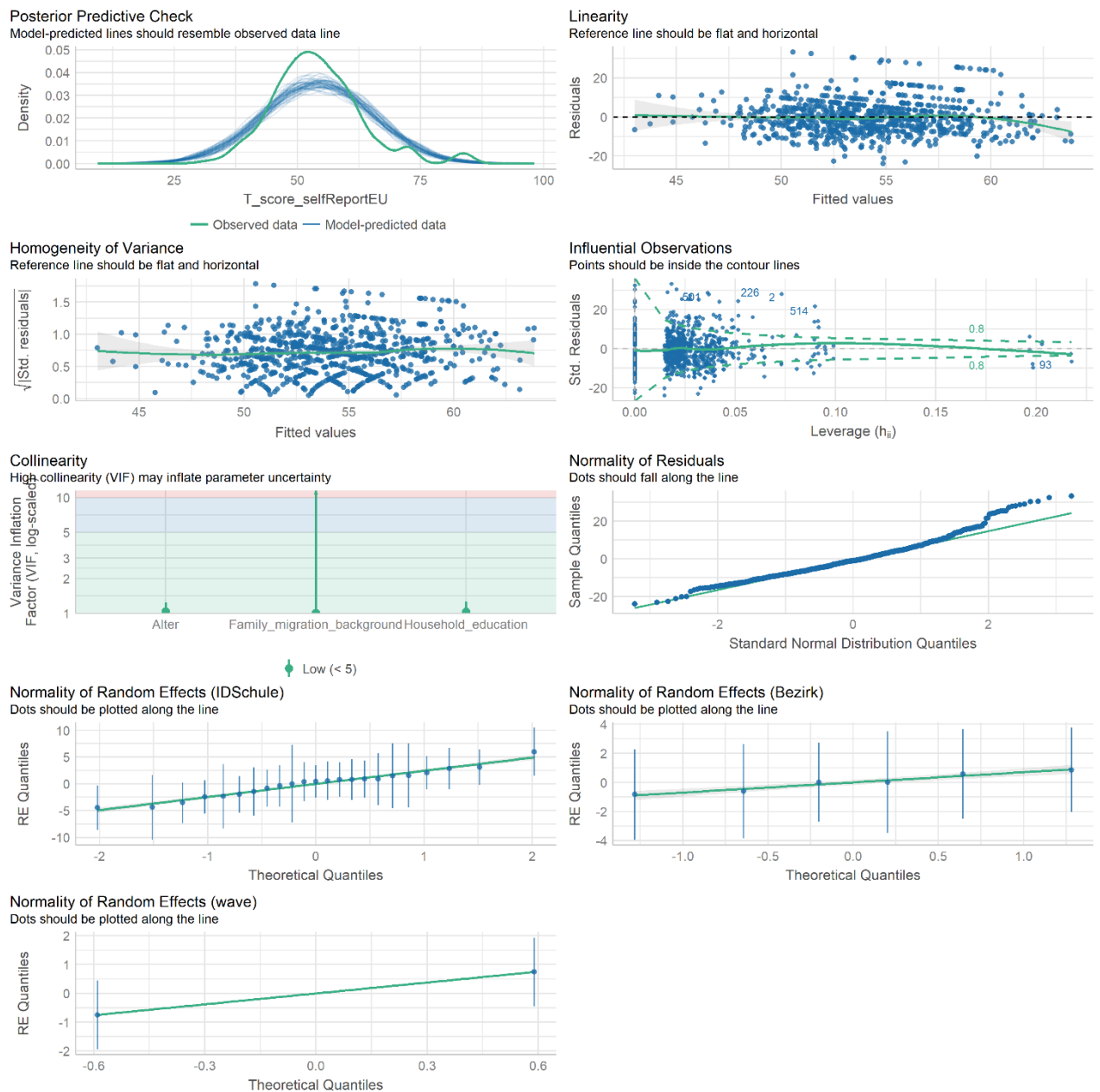

Supplement: S4 Fig — (PDF) [file pone.0302995.s009.pdf]
