# Supplementary material for: Health-related quality of life and impact of socioeconomic status among primary and secondary school students after the third COVID-19 wave in Berlin, Germany
Source: PLoS One. 2024 May 9;19(5):e0302995. doi: 10.1371/journal.pone.0302995 (PMC11081372; doi:10.1371/journal.pone.0302995)

S9 Fig. **HRQoL categories by time point, sex, and school type (primary vs. secondary) based on the IPW pseudo-population of 653 children.**

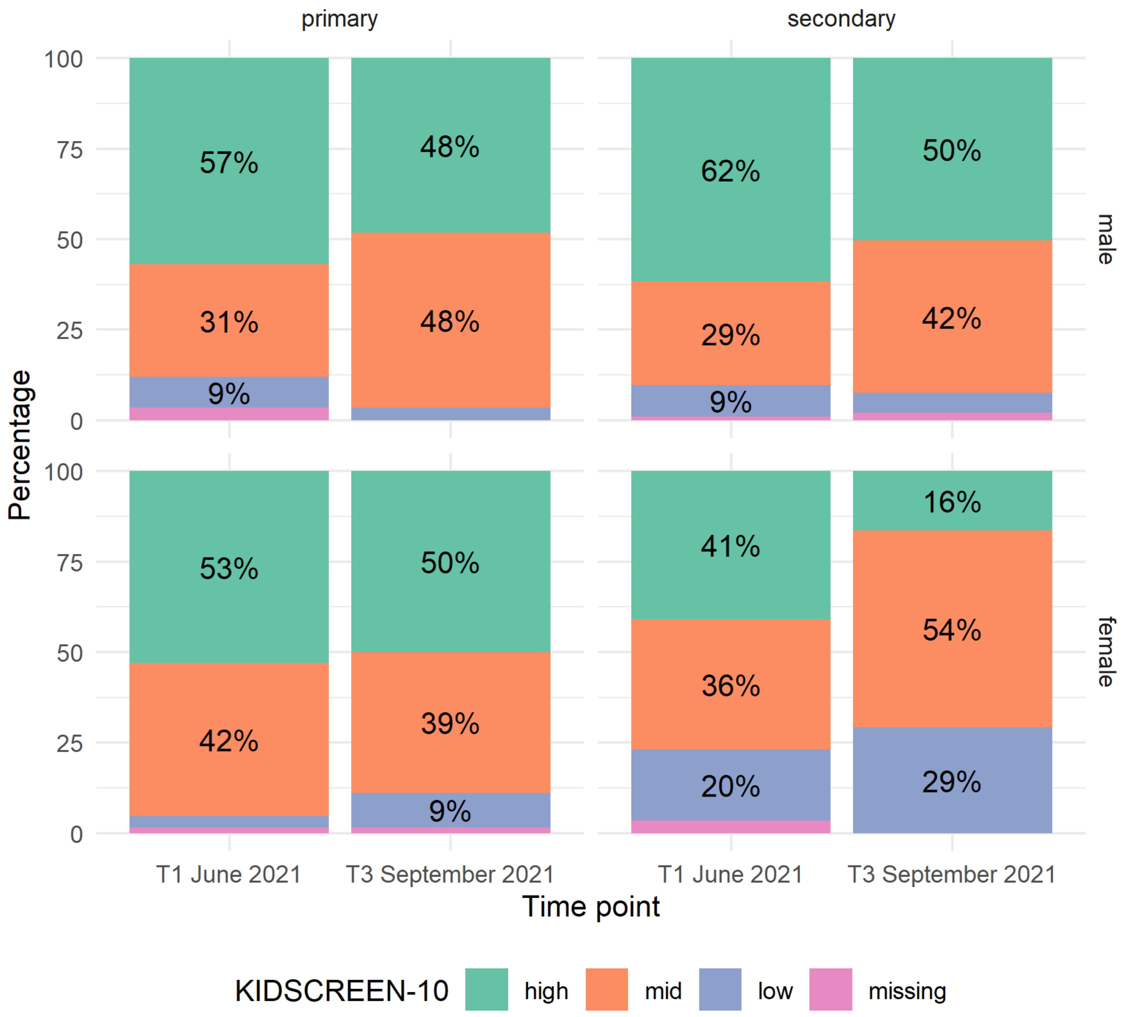

Supplement: S5 Fig — (PDF) [file pone.0302995.s010.pdf]
